# CADM1/TSLC1 Identifies HTLV-1-Infected Cells and Determines Their Susceptibility to CTL-Mediated Lysis

**DOI:** 10.1371/journal.ppat.1005560

**Published:** 2016-04-22

**Authors:** Kiruthika Manivannan, Aileen G. Rowan, Yuetsu Tanaka, Graham P. Taylor, Charles R. M. Bangham

**Affiliations:** 1 Section of Virology, Department of Medicine, Imperial College London, London, United Kingdom; 2 Department of Immunology, Graduate School of Medicine, University of the Ryukyus, Okinawa, Japan; University of Illinois at Chicago College of Medicine, UNITED STATES

## Abstract

Human T cell lymphotropic virus-1 (HTLV-1) primarily infects CD4^+^ T cells, causing inflammatory disorders or a T cell malignancy in 5% to 10% of carriers. The cytotoxic T lymphocyte (CTL) response is a key factor that controls the viral load and thus the risk of disease. The ability to detect the viral protein Tax in primary cells has made it possible to estimate the rate at which Tax-expressing infected cells are eliminated by CTLs in persistently infected people. However, most HTLV-1-infected cells are Tax^**–**^at a given time, and their immunophenotype is poorly defined. Here, we aimed to identify a cell-surface molecule expressed by both Tax^+^ and Tax^**–**^HTLV-1-infected cells and use it to analyse the CTL response in fresh peripheral blood mononuclear cells. Cell adhesion molecule 1 (CADM1/TSLC1) was the best single marker of HTLV-1 infection, identifying HTLV-1-infected cells with greater sensitivity and specificity than CD25, CCR4 or ICAM-1. CADM1^+^CD4^+^ T cells carried a median of 65% of proviral copies in peripheral blood. In a cohort of 23 individuals, we quantified the rate of CTL-mediated killing of Tax^+^ and Tax^**−**^CADM1^+^ cells. We show that CADM1 expression is associated with enhanced susceptibility of infected cells to CTL lysis: despite the immunodominance of Tax in the CTL response, Tax^+^CADM1^**–**^ cells were inefficiently lysed by CTLs. Upregulation of the CADM1 ligand CRTAM on CD8^+^ T cells correlated with efficient lysis of infected cells. Tax^**–**^CADM1^+^ cells were lysed at a very low rate by autologous CTLs, however, were efficiently killed when loaded with exogenous peptide antigen. High expression of CADM1 on most HTLV-1-infected cells in the face of enhanced CTL counterselection implies that CADM1 confers a strong benefit on the virus.

## Author Summary

Human T cell lymphotropic virus-1 (HTLV-1) infects white blood cells (CD4^+^ T cells) for the lifetime of the host. The immune response limits viral spread, and people with a weak immune response have a high risk of developing an aggressive blood cancer, or a condition involving irreversible spinal cord damage. Virus and host are engaged in a constant battle: virus proteins drive the host cell to divide or infect new cells. We know that the viral protein Tax is an important target of the immune response, and cells which produce Tax are killed quickly. Infected cells which do not produce Tax are difficult to detect, so we have no idea how quickly they are killed. In this paper we show that most infected cells have a host protein ‘CADM1’ on their surface. We measured killing of CADM1 cells and saw that Tax^+^CADM1^+^ cells are the only infected cells which are strongly targeted by the immune response. We also found that infected cells which did not have CADM1 on the surface escaped killing, showing that CADM1 aids in immune control of HTLV-1. These findings are an important step forward in our understanding of cellular turnover and immune control in chronic infection.

## Introduction

Human T-lymphotropic virus 1 (HTLV-1) is a retrovirus that predominantly infects CD4^+^ T cells. An estimated 10–20 million people are infected, with regions of high prevalence including Japan, Africa, the Caribbean and South America. The viral burden (proviral load, PVL) is strongly correlated with the risk of disease [1]. Between 1% and 6% of HTLV-1-infected individuals develop a T cell malignancy known as adult T cell leukemia/lymphoma (ATL), and an additional 2–3% suffer from a variety of inflammatory disorders, the most prevalent of which is HTLV-1 associated myelopathy/tropical spastic paraparesis (HAM/TSP). Although HTLV-1 was the first retrovirus observed to be pathogenic in humans, both effective treatment and a vaccine remain elusive.

HTLV-1 persists within an infected individual by infectious spread across the virological synapse and by mitotic replication of infected cells [2,3]; virus particles are usually undetectable in peripheral blood [4]. The proviral integration site imparts each infected T cell clone with a different pattern and intensity of viral gene expression [5,6]. Of these, Tax and HTLV-1 basic leucine zipper protein (HBZ), two regulatory proteins, play an important role in viral pathogenesis. The transcriptional transactivator, Tax, encoded in the positive strand in the regulatory (pX) region of the virus, controls the expression of viral proteins (Pol, Gag and Env) as well as many host genes [7]. The negative strand-encoded accessory gene HBZ can inhibit Tax function and modify transcription of various host genes [7].

The PVL of HTLV-1 reaches a stable (‘set-point’) level in each individual [8], which is maintained by the equilibrium between the proliferation of infected cells and their elimination by activated cytotoxic T lymphocytes (CTLs) [9,10]. Tax, which is immunodominant, is subject to strong selection pressure from the autologous CTL response [11], and a high lytic efficiency of HTLV-1-specific CTLs (defined as the rate of clearance of Tax^+^CD4^+^ T cells/% CD8^+^ T cells/day) is associated with low PVL and a low risk of HAM/TSP [12]. Tax expression in fresh peripheral blood mononuclear cells (PBMCs) is typically low in asymptomatic HTLV-1 carriers (ACs) and is silenced in ~50% of ATL clones [13–15]. In addition, CTL-selected Tax sequence variants are generally defective in their transactivating function [11], impairing expression of positive strand-encoded viral genes. In contrast, HBZ is persistently expressed at low levels under the control of SP1 transcription factors [16]. HBZ minimizes its exposure to the host immune response by virtue of low protein expression, low immunogenicity and poor binding to Class 1 MHC alleles [17,18]. Compared with Tax-specific CTLs, HBZ-specific CTLs are present at lower frequency in the peripheral blood and kill fewer HTLV-1-infected cells in vitro [17,18]. However, HBZ-specific CTLs appear to be more effective in controlling HTLV-1 during chronic infection in vivo [18,19].

A limitation of the previously described assay of anti-HTLV-1 CTL lytic efficiency [12] is that the identification of HTLV-1^+^ cells was defined by Tax expression, so the lysis rate of Tax^**−**^cells–including those that express the key CTL target antigen HBZ–was not quantified. To date, there has been no practicable means to differentiate an HTLV-1-infected cell from an uninfected cell without destroying it in the process, either by permeabilizing it to detect Tax or other viral proteins, or by extracting DNA to detect the viral genome. The aim of the present study was to identify the cell-surface phenotype that most efficiently distinguishes viable HTLV-1-infected cells from uninfected cells, to allow more accurate analysis of the cellular immune response to the virus and the frequency and phenotype of HTLV-1-infected cells.

Several activation markers, costimulatory receptors, chemokine and interleukin receptors and adhesion molecules are strongly expressed on HTLV-1-infected cells and serve as possible markers of HTLV-1 infection. These molecules include the IL-2 receptor α chain (CD25) [20], chemokine receptor 4 (CCR4/CD194) [21,22] and intercellular adhesion molecule 1 (ICAM-1/CD54) [23,24]. CD25 is induced by Tax, and the CD4^+^CD25^+^ population is typically ~90% HTLV-1-infected [20,25]. CCR4 is not directly induced by Tax [21], but CCR4^+^ cells appear to be preferentially infected by HTLV-1, perhaps because they are attracted to infected cells as a result of Tax-induced expression of CCL22 [26]. Yamano et al. (2009) recommended the use of both CD25 and CCR4 to isolate HTLV-1-infected cells with high purity [27]. ICAM-1, also induced by Tax, aids in the formation of the virological synapse [28–30]. However, although CD25, CCR4 and ICAM-1 are highly expressed in HTLV-1 carriers with a high viral load, they are also expressed on activated T cells and regulatory T-cells during inflammatory and immune responses to pathogens.

Cell adhesion molecule 1 (CADM1), also known as tumour suppressor in lung cancer 1 (TSLC1), nectin-like protein 2 (Necl-2), immunoglobulin superfamily member 4 (IGSF4), synaptic cell adhesion molecule (SynCAM) or spermatogenic immunoglobulin superfamily (SgIGSF) is a cell-surface molecule that has recently been proposed as a marker of malignant cells in ATL patients [31–33]. CADM1 is a nectin-like cell adhesion molecule of the immunoglobulin superfamily. It was initially identified as a tumour suppressor of a range of solid cancers (breast, ovarian, cervical and colorectal carcinomas, melanomas and neuroblastomas) [34]. Although CADM1 is expressed in a wide range of tissues, it is usually absent in leukocytes; typically 1% of T cells are CADM1^+^ [31,32,34–36]. CADM1 can form both homophilic and heterophilic interactions that stabilise cell-to-cell interactions. Outside-in CADM1 signalling can trigger the rearrangement of the actin cytoskeleton to alter cell polarity and motility [37]. CADM1 also interacts with another immunoglobulin superfamily cell-surface protein, Class-1 MHC-restricted T-cell associated molecule (CRTAM/CD355/Cytotoxic and Regulatory T Cell Molecule) on activated NK, CD8^+^ T and NKT cells. This interaction enhances NK cell-mediated cytotoxicity and IFN-γ secretion by CD8^+^ T cells [38,39]. CADM1 is consistently expressed on T-cells in ATL patients and in HTLV-1-transformed cell lines [31,32]; the percentage of CD4^+^CADM1^+^ cells in patients with ATL positively correlates with both the PVL and the frequency of morphologically abnormal lymphocytes [32]. Immunohistochemical staining of CADM1 in organs of NOD-SCID/γc^null^ mice transplanted with ATL cells showed a correlation between the level of CADM1 expression on ATL cells and the ability of the ATL cells to invade solid organs such as the liver, ovaries and lungs. It has been postulated that the homophilic adhesion promotes the growth of ATL cells in these organs, in which CADM1 expression is indigenous [31,40].

In the present study we evaluated the specificity of CD25, CCR4, ICAM-1 and CADM1 as markers of HTLV-1 infection in uncultured PBMCs of individuals with non-malignant HTLV-1 infection (ACs and patients with HAM). We then used the surface markers with the best combination of sensitivity and specificity to quantify and compare the rate at which autologous CTLs lysed Tax-expressing and non-expressing HTLV-1-infected cells.

## Results

### The frequency of CADM1^+^CD4^+^ T cells is positively correlated with HTLV-1 proviral load

**Fig 1:**
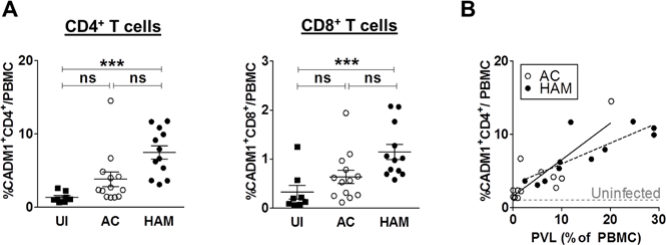
The frequency of CADM1^+^CD4^+^ T cells is positively correlated with proviral load. Uncultured PBMC from 32 individuals (uninfected (UI), n = 8; AC, n = 13; HAM, n = 11), were stained with antibodies specific for CADM1, CD25, ICAM-1, CCR4, CD4,CD8 and CD3 and analysed by flow cytometry. **(A)** Frequency of CADM1^+^CD4^+^ and CADM1^+^CD8^+^ T cells in uninfected individuals, ACs and patients with HAM. Bars denote mean ± SEM. Statistical analysis: Kruskal-Wallis test with Dunn post-test, 95% confidence interval (CI). Significant differences are highlighted, *** denotes p<0.0001. **(B)** Frequency of CADM1^+^CD4^+^ T cells versus PVL. Grey dashed line indicates median percentage of CADM1^+^CD4^+^ T cells/PBMC in 8 uninfected controls. Spearman correlation was performed for AC (p = 0.01, r_s_ = 0.66) and HAM (p = 0.003, r_s_ = 0.83). Continuous line represents linear regression of AC and dashed line that of HAM.

To identify a marker of HTLV-1-infected cells, we first analysed the expression of candidate cell-surface molecules (CADM1, CD25, CCR4 and ICAM-1) on uncultured PBMCs. PBMCs from 13 ACs, 11 patients with HAM and 8 uninfected individuals were analysed by flow cytometry, and the frequency of cells expressing the above markers within total PBMCs was calculated. Patients with HAM had significantly higher frequencies of CADM1^+^CD4^+^ and CADM1^+^CD8^+^T cells in PBMCs compared to uninfected individuals (Fig 1A). A positive correlation was observed between the frequency of CADM1^+^CD4^+^ T cells and the PVL, which accounts for the observation that the frequency of CADM1^+^CD4^+^ cells was greater in patients with HAM than in ACs (Fig 1B). While there was no significant difference between ACs and uninfected individuals, patients with HAM had a greater frequency of CD25^+^CD4^+^ and CCR4^+^ CD4^+^ T cells than uninfected individuals (S3A Fig). A similar pattern was observed in CD8^+^ T cells (S4 Fig). The frequency of total ICAM-1^+^CD4^+^ T cells in patients with HAM was higher than in ACs but was not significantly different from the frequency in uninfected individuals. Despite these differences, there was no significant correlation between the PVL and the frequency of CD25^+^, CCR4^+^ or ICAM-1^+^ cells (S3B Fig). The frequency of CCR4^+^ CD4^+^ and ICAM-1^+^ CD4^+^ T cells was significantly greater than the PVL, i.e. these markers are not specific to HTLV-1 infection.

### Tax expressing primary PBMCs are predominantly CADM1^+^

**Fig 2:**
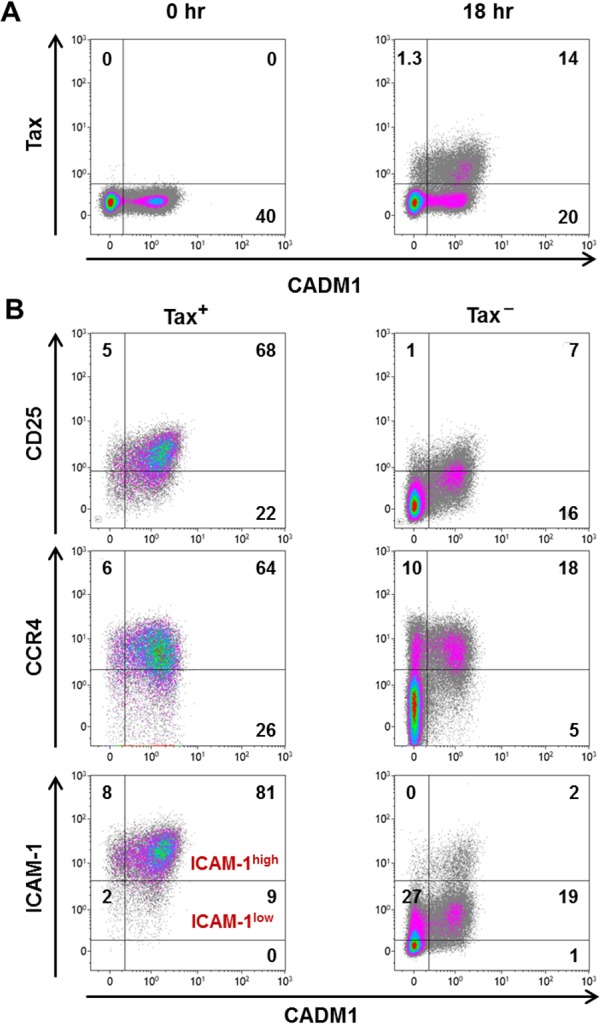
Concordance of Tax expression with putative markers of infected cells. PBMCs from the same cohort as in Fig 1 were cultured for 18 hours at 1 x 10^6^ cells/ml in the presence of 20 ng/ml CMA. Cells were stained with antibodies specific for CADM1, CD25, ICAM-1, CCR4 and CD4 followed by intracellular staining with an antibody specific for Tax, then analysed by flow cytometry. **(A)** Representative dot plots showing Tax and CADM1 expression in total CD4^+^ T cells in uncultured (0 hr) and cultured (18 hr) PBMCs (donor PVL 20.18%). Numbers indicate percentages of total live CD4^+^ cells. **(B)** Representative dot plots to compare the expression of CD25, CCR4 and ICAM-1 on Tax^+^ or Tax^**−**^CD4^+^ T cells. Numbers indicate percentages of the total Tax^+^CD4^+^ or Tax^**–**^CD4^+^ population respectively.

PBMCs from the same individuals were cultured for 18 hours to allow spontaneous viral gene expression in the presence of concanamycin A (CMA) to inhibit CTL-mediated lysis. Infected cells were detected by intracellular staining of the viral protein Tax. Tax expression was most frequent in cells expressing high levels of CADM1 (Fig 2A). There was no significant change in the frequency of CADM1^+^CD4^+^ T cells during incubation in vitro.

We quantified the frequency of expression of CD25, CCR4, ICAM-1 and CADM1 in Tax^+^ and Tax^**–**^CD4^+^ T cells. These data were used to calculate the sensitivity (% marker^+^Tax^+^/total Tax^+^) and the specificity (% Tax^+^marker^+^/total marker^+^) of detection of Tax^+^ cells by each respective surface marker (S5 Fig). The representative dot plots in Fig 2B show that most Tax^+^ cells expressed high levels of CADM1, CD25, CCR4 and ICAM-1. CCR4 and ICAM-1 were expressed at high frequencies on Tax^**–**^CADM1^–^ cells (Fig 2B). Thus, CCR4 and ICAM-1 had high sensitivity but lacked the specificity required for an optimal marker. However, a high intensity of ICAM-1 expression (ICAM-1^high^ gate, Fig 2B) identified Tax^+^ cells with high sensitivity (84%) and specificity (77%). The intensity of CD25 expression was correlated with that of Tax expression (S6 Fig), and CD25 was generally not expressed on CADM1^**–**^ cells. However, CD25 identified only a third of the Tax^**−**^infected population detected by CADM1 (Fig 2B), and the low intensity of expression of CD25 made it difficult to distinguish CD25^+^ cells from CD25^**–**^ cells.

### Sixty-five percent of the HTLV-1 load is carried is carried in CADM1^+^CD4^+^ T cells

**Fig 3:**
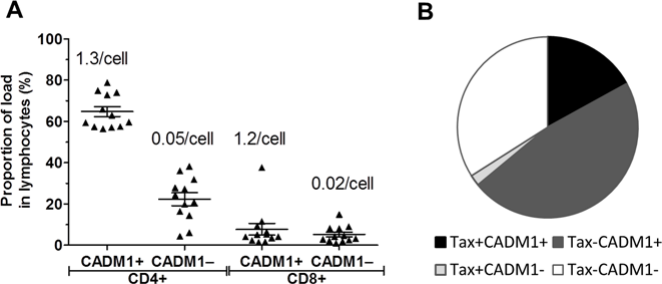
Proportion of proviral copies carried by CADM1^+^ lymphocytes. ***(A)*** *CADM1* ^*+*^ *CD4* ^*+*^ *T cells carry the bulk of the proviral load*. Purified CD4^+^ T cells from 12 individuals (AC, n = 6; HAM, n = 6,) were stained for surface markers and flow sorted as shown in S2 Fig. Genomic DNA was extracted from sorted populations and the PVL of each population was quantified. The frequency of each population in PBMC was used to calculate the relative contribution of each population to the proviral load. Bar denotes mean ± SEM. Numbers denote the median number of HTLV-1 copies detected per cell. ***(B)*** *CADM1 identifies ~65% of infected T cells*. Purified CD4^+^ T cells from 6 individuals were cultured for 16 hours, stained with antibodies specific for cell surface markers and Tax, then analysed by flow cytometry. Combining PVL data and the observed frequency of Tax^+^ cells, the median distribution of load among Tax^+/–^CADM1^+/–^CD4^+^ T cells was calculated. Pie chart represents the total proviral burden in CD4^+^ T cells. The white segment indicates infected cells that did not express Tax or CADM1.

Given that the frequency of CADM1^+^CD4^+^ T cells correlated with the proviral load, but exceeded the frequency of Tax-expressing cells, we wished to quantify the frequency of HTLV-1 infection in the CADM1^+^CD4^+^ cells. In order to do this, we flow-sorted uncultured live CD3^+^ T cells into the following subsets: CADM1^+^/^–^CD4^+^ and CADM1^+^/^−^CD8^+^ T cells, and quantified the number of HTLV-1 copies per cell (Fig 3A). We analysed 12 HTLV-1 infected donors (6 AC, 6 HAM) who had proviral loads between 3% and 31% of PBMC. Sorted CADM1^+^ lymphocytes had a consistently high proviral load: CADM1^+^CD4^+^ cells contained a median of 1.2 copies of HTLV-1 per cell, and CADM1^+^CD8^+^ cells contained a median of 1.3 copies (S8A Fig). In contrast, CADM1^–^CD4^+^ and CADM1^–^CD8^+^ cells carried a median of 0.05 and 0.02 copies per cell respectively. As proviral expression and virion production is negligible in chronically infected donors [4]; and patient derived infected primary T cell clones carry on average one proviral copy per cell [41], we interpret that each viral copy detected in this assay represents a single cell with one integrated provirus. Although CADM1^+^CD4^+^ and CADM1^+^CD8^+^ cells appear to be 100% infected, the frequency of CADM1^+^CD4^+^ cells was approximately tenfold higher than that of CADM1^+^CD8^+^ cells (S8B Fig). We calculated that a median of 65% of the proviral load in lymphocytes was carried in CADM1^+^ CD4^+^ cells (Fig 3A and S8 Fig); this proportion was consistent across all proviral loads tested (S8C Fig). In addition, an independent cell-sorting experiment gave a very similar median frequency of 64% (n = 6 individuals, S9 Fig). The next largest reservoir of infected cells were CADM1^–^CD4^+^ cells (median 22% of total load), which had a low per-cell proviral load (0.05 copies/cell), but were the most abundant population identified in this analysis (Fig 3 and S9B Fig). Although CADM1^+^CD8^+^ cells are heavily infected, their contribution to the proviral load was highly variable between donors (contributing between 1% and 37% of total proviral load, median contribution 7.7%); however, CADM1 remains a useful marker for enriching infected CD8^+^ cells.

If Tax positivity alone is used to identify cells carrying HTLV-1, on average 19% of infected cells would be detected, a majority of which (~90% of Tax^+^ cells) are CADM1^+^(Fig 3B). If CADM1 alone is used as a marker, we detect 65% of the infected cells, including both Tax^+^ and Tax^**–**^cells. Considering the facts that (i) Tax protein expression is usually undetectable in fresh PBMCs, (ii) Tax protein is expressed in only a proportion of infected cells even after in vitro culture and (iii) CADM1 identifies both Tax^+^ and Tax^**−**^infected cells with high purity even in fresh PBMCs, we conclude that CADM1 is the best single cell-surface marker for HTLV-1 infection identified to date.

### HTLV-1 Tax protein enhances CADM1 expression in response to cellular stimulation

**Fig 4:**
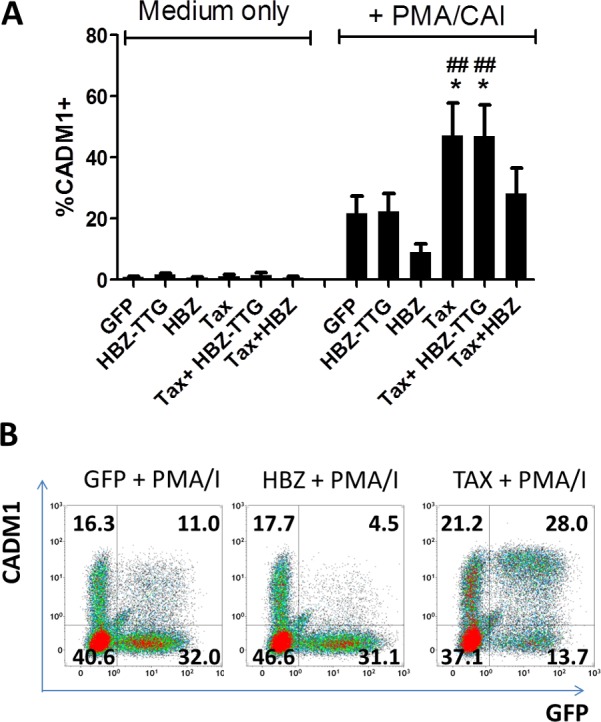
Modulation of CADM1 expression by HTLV-1 proteins. CEM cells were transfected with plasmids encoding Tax, HBZ-TTG, HBZ or GFP alone. Cells were cultured in the presence of medium only or in the presence of PMA/CAI. After 16h, cells were harvested and stained with a viability stain and anti-CADM1. The frequency of CADM1 expressing transfected cells was determined by gating on live GFP^+^ cells. (A) Data from three independent experiments. (B) Representative flow plots. Numbers indicate the percentage of live cells in each quadrant. Statistical analysis: Repeated measures Anova with Tukey post-test. *,p<0.05 versus GFP+PMA/CAI; ##, p<0.01 versus HBZ+PMA/CAI.

In order to further elucidate the role of HTLV-1 in the observed CADM1 expression, we tested whether a range of candidate HTLV-1 gene products could induce CADM1 expression on a CADM1 negative human T cell line. We observed that transfection with Tax or HBZ alone was not sufficient to induce CADM1 expression (Fig 4). As HBZ mRNA possesses biological activities distinct from HBZ protein, we also utilised a construct which expresses HBZ S1 with a mutation in the initiation codon:HBZ-TTG [42]. This construct did not induce CADM1 expression on unstimulated CEM cells. We hypothesised that HTLV-1 might modify the expression in response to an external stimulus. When stimulated with PMA/CAI, a fraction of CEM cells expressed CADM1. Tax transfection induced a twofold increase in the number of cells expressing CADM1 (Fig 4A). The intensity of CADM1 expression by Tax-transfected PMA/CAI stimulated cells was also significantly higher than the intensity of GFP transfected cells which were stimulated in the same way (Fig 4B). HBZ protein, but not HBZ RNA, consistently downregulated PMA/CAI induced CADM1 (3/3 biological replicates), and could reverse Tax-mediated enhancement of CADM1 expression (3/3 biological replicates).

### CADM1 expression is associated with efficient CTL-mediated killing of infected cells

**Fig 5:**
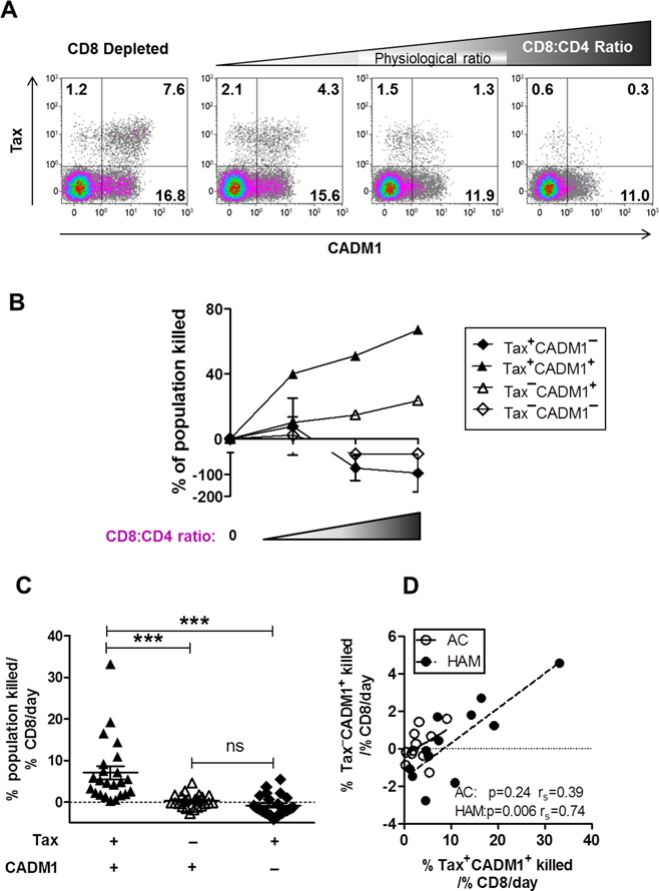
CADM1^+^ cells are killed more efficiently than CADM1^–^ cells by autologous CTLs. CD8^+^ T cells were depleted from PBMCs from 23 HTLV-1 infected donors (AC, n = 11; HAM, n = 12) and added back at a range of CD4^+^:CD8^+^ ratios. Cells were co-cultured for 18 hours, after which the frequency of Tax and/or CADM1 expressing cells was assayed by flow cytometry. **(A)** Representative dot plots showing Tax and CADM1 expression by live CD4^+^ T cells in a single individual. Numbers indicated in dot plots refer to percentage of live CD4^+^ T cells in each respective sample. **(B)** The absolute frequency of each subset in CD8^+^ depleted fraction was used as a baseline to calculate the percentage of each subset killed at each CD4^+^:CD8^+^ ratio. **(C)** The rate of lysis of each subset / %CD8^+^ T cells/day was calculated as described in materials and methods. Bars represent mean ± SEM. Statistical analysis: Friedman test with Dunn post-test, 95% CI. *** denotes p<0.0001 **(D)** The rate of lysis of Tax^+^CADM1^+^ cells significantly correlated with that of Tax^**–**^CADM1^+^ cells. Statistical analysis: Spearman correlation. Solid linear regression line, ACs; Dashed linear regression line, HAM.

Previous work indicates that an efficient Tax-specific CTL response is associated with a lower PVL [12,43] and a lower risk of HAM. However, most infected cells do not express Tax ex vivo or in short-term culture in vitro. MacNamara et al. (2010) showed that an HBZ-specific CTL response was associated with effective control of HTLV-1 in vivo [18]. We therefore wished to measure the rate at which Tax^**–**^CADM1^+^ cells are killed by CTLs. We used the infected cell elimination assay [12] to quantify the selective pressure exerted by autologous CD8^+^ T cells on the different cell subpopulations in vitro, using samples from 23 infected individuals (11 ACs and 12 patients with HAM) Fig 5A and 5B. Incorporation of counting beads in the assay enabled us to quantify the cells of each different phenotype and hence calculate the rate at which each population was lysed by CTL (Fig 5C).

The rate of CTL lysis of Tax^+^CADM1^+^ cells was significantly higher than that of any other population identified (%target population / % CD8^+^ T cell per day, Fig 5C), with a median of 45% of Tax^+^CADM1^+^ cells lysed over 18 hours at each individual's physiological CD4:CD8 ratio. The rate of lysis of Tax^**–**^CADM1^+^ and Tax^+^CADM1^–^ populations was not significantly different from zero, however, the rate of lysis of Tax^+^CADM1^+^ cells was significantly correlated with that of Tax^**–**^CADM1^+^ cells (Fig 5D) in HAM patients. The mean CTL lytic efficiency in the ACs in this cohort was lower than in previous studies, which may explain the observed lack of correlation in this group. Finally, although the kinetics of viral gene expression in CADM1^–^ cells was indistinguishable from CADM1^+^ cells (S7 Fig), Tax^+^CADM1^–^ cells, which were present at the lowest frequency, consistently increased in frequency on co-culture with CD8^+^ T cells (from a median of 0.6% of live CD4^+^ cells in CD8 depleted fraction to a median of 0.83% of live CD4^+^ cells at the physiological CD4:CD8 ratio). Thus, although the Tax^+^CADM1^–^ cells expressed the immunodominant CTL target antigen Tax, the absence of CADM1 was associated with escape from CTL lysis.

### The frequency of CRTAM^+^CD8^+^ T cells correlates with rate of lysis of Tax^+^CADM1^+^ cells

**Fig 6:**
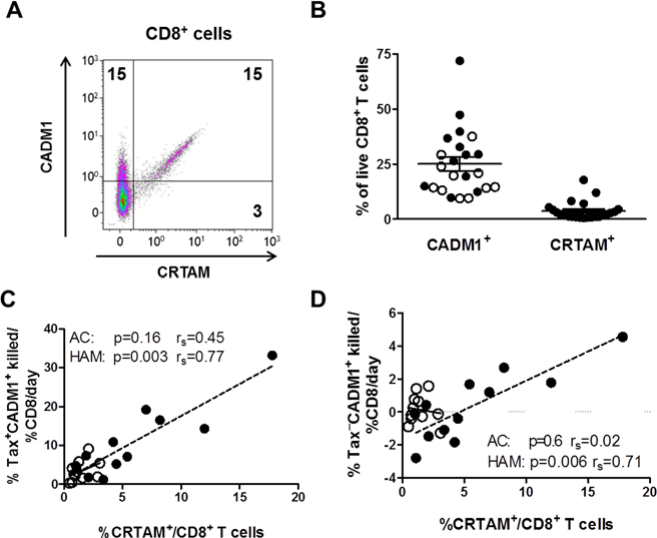
The frequency of CRTAM^+^CD8^+^ T cells is positively correlated with the rate of lysis of Tax^+^CADM1^+^ cells. CD8^+^ T cells cultured at the physiological CD4^+^:CD8^+^ ratio in the autologous CTL lysis assay (Fig 4) were stained with antibodies specific for CADM1 and CRTAM (AC, n = 11, empty circles; HAM, n = 12, closed circles). (A) Representative dot plot of CADM1 and CRTAM on live CD8^+^ T cells. Numbers indicate percentage of cells within gate. (B) Total frequencies of CADM1^+^ and CRTAM^+^ cells within live CD8^+^ T cells. Bars represent mean and SEM. The frequency of CRTAM^+^CD8^+^ T cells was positively correlated with the rate of lysis of the Tax^+^CADM1^+^ T cells (C) and Tax^**–**^CADM1^+^ T cells in HAM patients (D). Continuous line, regression line for ACs; dashed line, regression line for HAMs.

As CADM1 expression on the surface of the infected cell is associated with its susceptibility to CTL-mediated lysis, we quantified the expression of CADM1 and CRTAM on the surface of CD8^+^ T cells from individuals infected with HTLV-1. As CRTAM is not expressed ex vivo, and is upregulated on antigen recognition [38] we quantified CRTAM after autologous CD8^+^ T cells were cultured with HTLV-1-infected cells at each individuals’ physiological CD4^+^:CD8^+^ ratio for 18 hours. As previously reported by others, a majority of the CRTAM^+^CD8^+^ T cells co-expressed CADM1 (Fig 6). The frequency of CADM1^+^ CD8^+^ cells always exceeded the frequency of CRTAM^+^CD8^+^ with a median of 21% of live CD8^+^ expressing CADM1 and 2% CRTAM post incubation, and the frequency of CRTAM^+^CD8^+^ but not CADM1^+^CD8^+^ T cells positively correlated with the rate of lysis of the Tax^+^CADM1^+^ cells. These data indicate that after coincubation with autologous targets at the physiological CD4^+^CD8^+^ ratio, activated antigen-specific CD8^+^ T cells from HTLV-1-infected patients express the ligand for CADM1.

### CADM1 determines the susceptibility of infected cells to CTL-mediated lysis

**Fig 7:**
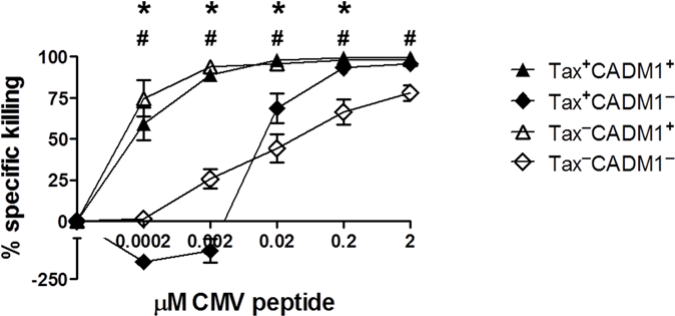
CADM1^+^CD4^+^ T cells are highly susceptible to CTL-mediated lysis. Purified CD4^+^ T cells from 6 HLA-A*02^+^ infected individuals (AC, n = 3; HAM, n = 3) were placed in culture to allow viral protein expression. After 6h incubation, cells were loaded with 0–2μM of an HLA-A*02-restricted CMV peptide, pp65 (NLVPMVATV) and co-cultured with a pp65-specific CTL clone at an E:T ratio of 1:1 for a further 12 hours. Cells were then stained for surface and intracellular antigens and analysed by flow cytometry. The median specific killing of Tax^+^ and CADM1^+^ cells was calculated after subtracting the background in the 0 μM peptide sample. Bars denote mean and SEM. Wilcoxon signed rank test was performed to compare populations. * denotes p = 0.03, Tax^+^CADM1^+^ vs. Tax^+^CADM1^–^; # denotes p = 0.03, Tax^**–**^CADM1^+^ vs.Tax^**–**^CADM1^–^.

To test whether expression of CADM1 is associated with enhanced target cell killing by CTLs specific to an antigen unrelated to HTLV-1, we pulsed primary HLA-A*0201^+^CD4^+^ T cells from HTLV-1^+^ donors with a HLA-A*0201-restricted CMV peptide and co-incubated them with a CMV-specific CTL clone. Even at very low concentrations of peptide, CADM1^+^CD4^+^cells were highly sensitive to CTL-mediated lysis: indeed, the Tax^**–**^CADM1^+^ cells were more significantly susceptible to CTL lysis than the Tax^+^CADM1^+^ cells at 0.0002μM and 0.002μM peptide (Fig 7). In contrast, the smaller Tax^+^CADM1^–^ population escaped lysis and increased in frequency at lower peptide concentrations, but became increasingly susceptible to lysis at peptide concentrations greater than 0.002μM. These data indicate that CADM1 expression is more important than Tax-induced factors (for example, ICAM-1) in determining a cell’s susceptibility to CTL-mediated lysis: CADM1^–^CD4^+^ T cells required at least 100–1000 fold more antigen presentation on the surface to elicit the same level of CTL lysis observed in the CADM1^+^CD4^+^ population. The CTL clone used in this assay was CADM1^–^CRTAM^−^in the absence of stimulation, but upregulated both CADM1 and CRTAM after anti-CD3 stimulation.

## Discussion

Since a typical HTLV-1-infected individual has between 10^4^ and 10^5^ distinct clones of infected T cells [44], it is unlikely that a single cell-surface marker can identify all the infected cells in an individual, owing to between-clone variation in proviral expression. Nevertheless, the results presented here show that expression of the single surface marker CADM1 identifies approximately 70% of HTLV-1-infected cells (CD4^+^ and CD8^+^) in the peripheral blood.

Although most HTLV-1-infected cells were CCR4^+^ and ICAM-1^+^, these markers lacked the specificity required of a single marker for infected cells: the frequency of CCR4^+^ and ICAM-1^+^ cells significantly exceeded the PVL in most HTLV-1-infected individuals. High-intensity ICAM-1 expression efficiently identifies Tax-expressing cells after incubation of PBMCs of infected individuals in vitro for 6 hours; however, ICAM-1 is not upregulated on Tax^**−**^cells. Yamano et al. proposed the use of CD25 alone or in combination with CCR4 to isolate infected cells [20,27]. While our data confirm that a high proportion of CD4^+^CD25^+^CCR4^+^ cells are infected, these cells accounted for a small proportion of the proviral load. The use of CADM1 alone in fresh cells not only identified an average of two-thirds of the cells identified by the use of CD25 and CCR4 but also detected a much larger population of infected CD25^**–**^CCR4^+^ cells. That is, the use of CADM1 alone identified twice the number of infected cells identified by the combination of CD25 and CCR4. CADM1 also identified a population of CD8^+^ cells which were heavily infected with the virus.

CADM1 has been reported as a surface marker of ATL cells [31–33]. The results of the present study extend the use of CADM1 as a marker of HTLV-1 infection to those without ATL. Kobayashi et al. reported that CADM1^+^ cells were frequently HTLV-1 infected and that the percentage of CADM1^+^ cells (both CD7^dim^ and CD7^**–**^) reflected disease status [45,46]. These authors also suggested that the appearance of CADM1^+^ cells is a marker of progression to ATL. However, we detected CADM1 expression in the PBMCs of all ACs and patients with HAM tested. We hypothesise that although ATL cells may arise from CADM1^+^ cells (expanded clones in ATL patients express CADM1 [31]), the expression of CADM1 in ACs or patients with HAM does not presage the onset of ATL. Rather, our data indicate that the frequency of CADM1^+^ cells reflects the PVL; a higher PVL is associated with a higher risk of ATL.

It remains unknown what induces CADM1 expression in HTLV-1-infected cells. There are conflicting reports on whether Tax induces CADM1. While we and others (Nakahata et al. 2012) failed to induce CADM1 in CEM, JPX-9, MOLT4 and 293T cells on introduction of a Tax expression vector [32], Pujari et al. (2015) found elevated levels of CADM1 in murine embryonic fibroblasts and Jurkat T cells upon lentivirus-mediated Tax expression [47]. Although we observed that Tax^+^ cells are mostly CADM1^+^ and Tax^+^CADM1^+^ cells express on an average 2–3 fold higher intensity of CADM1 than Tax^**–**^CADM1^+^ cells (S7 Fig), we conclude that Tax alone does not induce CADM1 in resting primary PBMCs, for the following reasons: (i) when PBMCs were cultured for 18 hours in vitro, the percentage of CADM1^+^CD4^+^ T cells did not increase with Tax expression; (ii) a significant proportion of CADM1^+^ cells did not express Tax; (iii) Tax is silenced, mutated or deleted in ~65% of ATL cases [13–15] whereas CADM1 is expressed in virtually all cases [31,33]. We observed that HBZ protein could downregulate CADM1 expression in response to an external stimulus, even in the presence of Tax. We postulate that the ability of the virus to modulate the level of CADM1 expression is advantageous to viral persistence. Indeed, it has recently been demonstrated that CADM1 plays an important role in the constitutive NF-κB activation observed in HTLV-1-infected cells [47].

To analyse the CD8^+^ T-cell response to HTLV-1-infected cells, we used a modified version of the functional assay previously described [12]. The modifications provided two significant advantages over the original assay: (i) the use of counting beads made it possible to enumerate cells in several target populations (defined by different phenotypes), and thus to quantify the rate at which they are killed by CTLs. (ii) The use of CADM1 made it possible to estimate the rate of lysis of the Tax^**–**^HTLV-1-infected population.

We observed that Tax^+^CADM1^+^ cells were lysed by autologous CTL more efficiently than Tax^+^CADM1^–^ and Tax^**–**^CADM1^+^ cells in all individuals assayed. Since the majority of the Tax^+^ cells are CADM1^+^, this result agrees with previous observations that cells expressing the immunodominant Tax protein are efficiently lysed by autologous CTLs [12,43]. The Tax^**–**^CADM1^+^ cells were indeed lysed at a lower rate than Tax^+^CADM1^+^ cells. Because Tax controls the expression of the plus-strand encoded proteins Pol, Gag and Env, it is likely that, with the exception of HBZ, the level presentation of epitopes from other viral genes is lower in Tax^**−**^infected cells than in Tax^+^ infected cells. Weak immunogenicity and low levels of presentation of HBZ peptides [17] contribute to the lower rate of lysis of this population. Surprisingly, a small proportion of Tax^+^CADM1^–^ cells were found to evade lysis. Thus, we propose that CADM1 facilitates CTL recognition and lysis of the infected cell and that Tax expression alone is not sufficient. Our results indicate that the efficiency of CTL killing of the Tax^+^CADM1^+^ population is predictive of the efficiency of killing of the Tax^**–**^CADM1^+^ population; i.e., individuals with a higher rate of lysis of the Tax^+^CADM1^+^ cells also had a higher rate of lysis of the Tax^**–**^CADM1^+^. This lower rate of clearance of the Tax-negative infected cells may be sufficient to maintain the equilibrium in vivo, because these cells do not undergo Tax-induced proliferation. As previously reported [12], the rate of lysis parameter at a given PVL was greater in patients with HAM than in ACs.

Using a CMV-specific CTL clone, we observed that CADM1^+^ cells, whether Tax^+^ or Tax^**–**^, were more susceptible than CADM1^–^ cells to CTL-mediated lysis, even at very low concentrations of antigen. We conclude that Tax-induced host proteins make little contribution to the cells’ susceptibility to CTL-mediated lysis. The CADM1^–^ cells, even those that express Tax, have very low susceptibility to CTL lysis unless they are loaded with artificially high concentrations of the peptide. Hence, we conclude that CADM1 is a major determinant of the susceptibility of the cell to CTL-mediated lysis. Rowan et al. (2014) reported that Tax-expressing CD4^+^ T cells were preferentially killed by CTLs of unrelated specificity [17]. As Tax^+^ cells are dominated by those of the CADM1^+^ phenotype, we attribute the preferential lysis of Tax^+^ cells in this experiment by CTLs to the presence of CADM1 on their surface. The susceptibility to lysis of the smaller Tax^**–**^CADM1^+^ and Tax^+^CADM1^–^ populations observed in our assay are likely to have been masked by the larger proportions of Tax^**–**^CADM1^–^ cells and Tax^+^CADM1^+^ cells respectively.

CD8^+^ T cells, when cultured with HTLV-1-infected CD4^+^ T cells, expressed both CADM1 and CRTAM. Although the CD8^+^ T cells had a much greater frequency of expression of CADM1 than CRTAM, the frequency of CADM1 expression bore no relationship to the CTL efficiency, and in fact represents the frequency of HTLV-1 infected CD8^+^ T cells. However, the frequency of CRTAM expression on CD8^+^ T cells was associated with high rates of CTL lysis. We propose that the interaction of CADM1 with CRTAM stabilises and prolongs the contact between the infected cell and the CTL and lowers the threshold of CTL activation.

To summarise, efficient CTL-mediated lysis of HTLV-1-infected cells is associated with the expression of CADM1 on the target cell and the high affinity ligand CRTAM on the effector CTL. Since CADM1 expression is tolerated in the face of enhanced CTL surveillance of HTLV-1-infected cells, we infer that CADM1 expression plays an important role in the persistence of HTLV-1.

## Materials and Methods

### Ethics statement

All donors attended the National Centre for Human Retrovirology (Imperial College Healthcare NHS Trust, St Mary's Hospital, London) and gave written informed consent in accordance with the Declaration of Helsinki, with the approval of UK National Research Ethics Service (15/SC/0089).

### Primary cells

Samples were donated by 29 ACs, 1 HTLV-1^+^ subject with polymyositis (P), 30 patients with HAM/TSP and 8 uninfected individuals (S1 Table). PBMCs were isolated from whole blood by density-gradient centrifugation using Histopaque-1077 (Sigma) and cryopreserved in FBS (Invitrogen) containing 10% dimethylsulphoxide (Sigma). Genomic DNA was extracted from unfixed PBMCs using the DNeasy kit (Qiagen) according to the manufacturer’s protocol. The lysis step was extended from 10 minutes at 56°C to 16 hours at 62°C when extracting DNA from formaldehyde-fixed cells. To quantify the PVL, a series of dilutions of genomic DNA starting from 5ng/μl was subjected to real-time quantitative PCR (qPCR) with the following primers: Tax: SK43 5’-CGGATACCCAGTCTACGTGT-3’ and SK44 5’-GAGCCGATAACGCGTCCATCG-3’; GAPDH: GAPDHF 5’- AACAGCGACACCCATCCTC-3’ and GAPDHR 5’- CATACCAGGAAATGAGCTTGACAA-3’. qPCR was performed using the QuantStudio 7 Flex real-time PCR system (Life technologies) with the standard Fast SYBRgreen (Life technologies) thermal cycle protocol. A patient-derived infected CD4^+^ T cell clone with a mapped single integrated provirus was used as a standard reference [41].

### Flow cytometric analysis of marker expression

PBMCs were analysed either uncultured or after culturing for 18 hours at a density of 1 x 10^6^ cells/ml in RPMI-1640 (Sigma) containing 10% FBS, 2mM L-glutamine (Sigma), 50 U/ml penicillin, 50μg/ml streptomycin (RPMI10, Sigma), supplemented with 20nM concanamycin A (CMA, Calbiochem). This incubation allowed the onset of spontaneous Tax expression while CD8^+^ T-cell degranulation was inhibited by concanamycin A. Cells were washed once with PBS (Sigma), then stained with a fixable viability dye (Live/Dead blue or Live/Dead near infrared, Molecular Probes) at 1μl/ml for 5 minutes. All steps were carried out at room temperature unless stated otherwise. Cells were washed with FACS buffer (PBS 7% normal goat serum, Sigma). Then they were stained for 20 minutes with antibodies specific for surface markers, including CD3-Qdot605 (clone UCHT1, Molecular Probes); CD4-eFluor450 (RPA-T4, eBioscience); CD8-AF700 (LT8, AbD Serotec); CCR4-PerCP-Cy5.5 (TG6/CCR4, Biolegend); ICAM-1-PE (HA58, Biolegend); CD25-APC (M-A251, BD) and CADM1 (3E1, MBL) biotinylated using EZ-Link Micro Sulfo-NHS-LC-Biotinylation Kit (Thermo Fisher Scientific). Unbound antibodies were removed by washing with FACS buffer. The cells were then stained for 10 min with Streptavidin-PECy7 (0.4μl/100μl, Biolegend). After another wash with FACS buffer, the cells were fixed with fixation/permeabilisation buffer (FoxP3 buffer set, eBioscience) for 30 minutes. The cells were then washed with the permeabilisation buffer and stained for the intracellular viral protein Tax for 25 minutes using the LT-4 antibody conjugated to AF488 (Y. Tanaka). Finally the cells were washed and resuspended in PBS, after which they were acquired using a BD LSRFortessa. Data was analyzed using Kaluza software (Beckman Coulter). The gating strategy is illustrated in S1 Fig.

### Flow sorting CADM1^+^ populations

PBMC from twelve HTLV-1 infected donors were stained with a viability stain and antibodies specific for CD4, CD8, CD3, CADM1, CD25 and CCR4 as described. Cells were fixed with 2% paraformaldehyde for 20 min, after which live CD3^+^ CADM1^+/–^CD4^+^ and CADM1^+/–^CD8^+^ cells were sorted with a BD FACSAria III as outlined in S2 Fig. Genomic DNA was extracted from both unfractionated PBMC and sorted samples, and the number of proviruses present in each fraction was estimated by qPCR. The frequency CADM1^+^ and CADM1^–^ cells in uncultured CD3^+^ cells was used to calculate the proportion of proviral load which was carried in each population of cells in each individual.

### Electroporation of CEM cells

CEM cells were electroporated with 2 μg of the following plasmids: GFP-Tax [48], HBZ-IRES- GFP (pCMV.IRES.GFP.Myc(x2)_HBZ.SP1; [49]), and/or GFP alone (pCMV.IRES.GFP.Myc(x2); Clontech), or 1 μg HBZ-TTG (pME18Sneo-HBZ TTG; [42]) plus 1 μg GFP or–GFP-Tax where indicated. Electroporation was carried out using a nucleofector 1 device (program A030) which routinely gave 20–40% transfection efficacy with ~95% viability. Post electroporation cells were placed in warm RPMI 10% FCS at a density of 3x10^5^/ml in the presence or absence of 10 ng/ml phorbol 12-myristate 13-acetate (PMA) and 0.5 μg/ml calcimycin (CAI, Sigma). After 16h culture, cells were harvested, stained with a viability stain and biotinylated anti-CADM1, followed by Streptavidin-PEDazzle (Biolegend). Cells were analysed by flow cytometry within 1 h.

### Infected cell elimination assay (by autologous CTL)

CD8^+^ T cells were depleted from PBMCs by magnetic cell separation (Miltenyi Biotech), then mixed with the CD8-depleted fraction at a range of ratios (including the individual’s physiological CD4^+^:CD8^+^ ratio) and co-cultured for 18 hours at a density of 1 x 10^6^ cells/ml in RPMI10 supplemented with 20μg/ml DNase (Sigma). All samples were assayed in duplicate. Following co-culture, 10% of the cells in each tube were set aside for estimation of absolute cell counts and the remainder were stained as described above with a viability stain, anti-CD3-BV510 (UCHT-1, Biolegend), -CD4-BV605 (RPA-T4, Biolegend), -CRTAM-PE (Cr24.1, Biolegend), -CD8-AF700, -CADM1–Biotin and -Tax-Cy5. For absolute cell counts, cells were stained with CD4-BV605 and CD8-AF700 for 30 min and, without washing, fixed with 2% paraformaldehyde (Sigma) for 30 minutes. A fixed number of CountBright absolute counting beads (Molecular Probes) were added to each sample before flow cytometric analysis. Thus, the absolute number of CD4^+^ and CD8^+^ T cells per tube was calculated and used to calculate the rate of clearance of target CD4^+^ T cells per percentage CD8^+^ T cells per day, as described in Asquith et al 2005 [12].

### CMV CTL clone

A cytomegalovirus (CMV)-specific CD8^+^ T cell clone (a gift from Tao Dong) that recognises an HLA-A*0201-restricted peptide, PP65 (NLVPMVATV), was expanded by co-culturing with gamma-irradiated (3000 rads) PBMCs in RPMI10 containing 50 μg/ml phytohemagglutinin (Roche) and 100 IU/ml IL-2 (Promocell). The cells were fed with fresh medium and IL-2 every 3–4 days. The CTLs were used in the susceptibility assay on day 17 post stimulation.

### Assay of target cell sensitivity to CTL

CD4^+^ T cells were isolated from HLA-A*0201^+^ PBMCs using magnetic cell separation (Miltenyi Biotech) and cultured for 6 hours (1 x 10^6^ cells/ml in RPMI10 containing 20μg/ml DNase), to allow Tax expression. The cells were then loaded with the CMV peptide pp65 (Think Peptides) at a range of concentrations (0–2 μM). CMV-specific CTLs were added to the peptide-loaded CD4^+^ cells at an effector:target ratio of 1:1 and co-cultured for 12 hours. All samples were assayed in duplicate. A small portion of cells was used to determine absolute cell counts and the remainder were stained with a viability stain, anti-CD3-BV510, -CD4-BV605, -CRTAM-PE, -CD8-AF700, -CADM1–Biotin and -Tax-Cy5.

## Supporting Information

S1 Table Donor information. (XLSX)

S1 Fig Flow cytometric gating strategy for cell surface analysis. (TIF)

S2 Fig Flow cytometric gating strategy for cell sorting experiments. (TIF)

S3 Fig CD25, CCR4 and ICAM-1 expression on uncultured CD4^+^ cells. **(A)** The median frequency of cells in each group of patients. **(B)** PVL versus frequency of cells in each subset.
(TIF)

S4 Fig CD25, CCR4 and ICAM-1 expression on uncultured CD8^+^ cells. (TIF)

S5 Fig Sensitivity and specificity with which each surface marker detects Tax expressing cells. (TIF)

S6 Fig CD25^+^CADM1^+^ cells are more likely to express Tax than CD25^–^CADM1^+^ cells. **(A)** Representative dot plot. **(B)** The proportion of CADM1^+^CD25^+^ and CADM1^+^CD25^**–**^ cells that express Tax after 16h culture.
(TIF)

S7 Fig Kinetics and intensity of Tax expression in CADM1^+^/^–^CD4^+^ T cells. **(A)** Frequency of live CD3^+^CD4^+^ cells expressing Tax. **(B)** Intensity of Tax expression by live CAMD1^+^/^–^CD3^+^CD4^+^ cells
(TIF)

S8 Fig Distribution of proviral load in PBMC. **(A)** Copies of HTLV-1 per cell for each individual. **(B)** Frequency of T cell subsets in PBMC. **(C)** Proportion of load carried in CADM1^+^ lymphocytes versus proviral load in PBMC.
(TIF)

S9 Fig Median PVL and proportion of cells expressing Tax in CD4^+^ cells sorted on the basis of CADM1, CCR4 and CD25 expression. **(A)** Sorting strategy. **(B)** PVL of total CD4^+^ T cells and sorted populations. **(C)** The proportion of load carried by CADM1 or Tax expressing CD4^+^ cells.
(TIF)
