# Supplementary figures and images for: CADM1/TSLC1 Identifies HTLV-1-Infected Cells and Determines Their Susceptibility to CTL-Mediated Lysis
Source: PLoS Pathog. 2016 Apr 22;12(4):e1005560. doi: 10.1371/journal.ppat.1005560 (PMC4841533; doi:10.1371/journal.ppat.1005560)

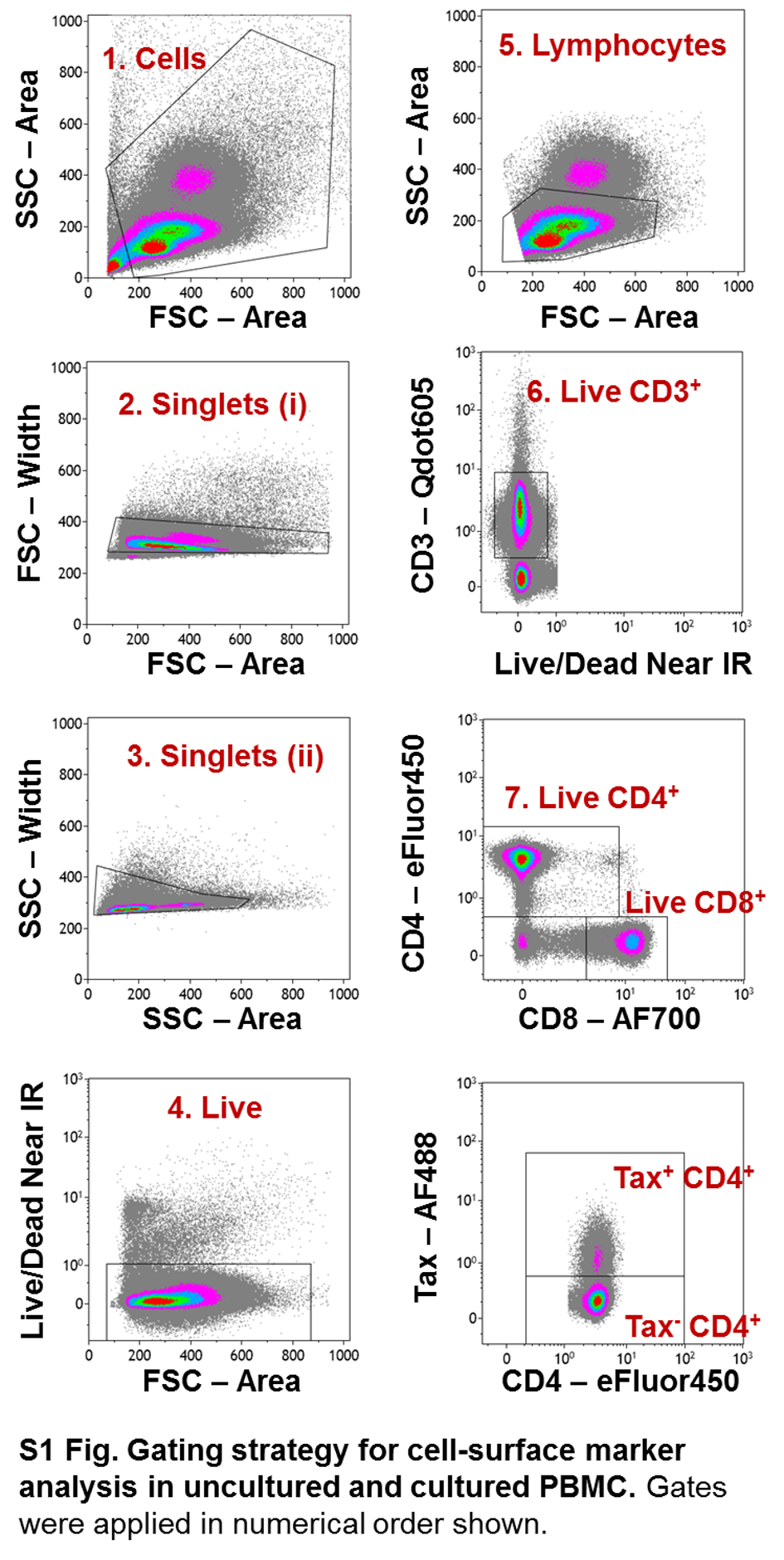

Supplement: S1 Fig — (TIF) [file ppat.1005560.s002.tif]

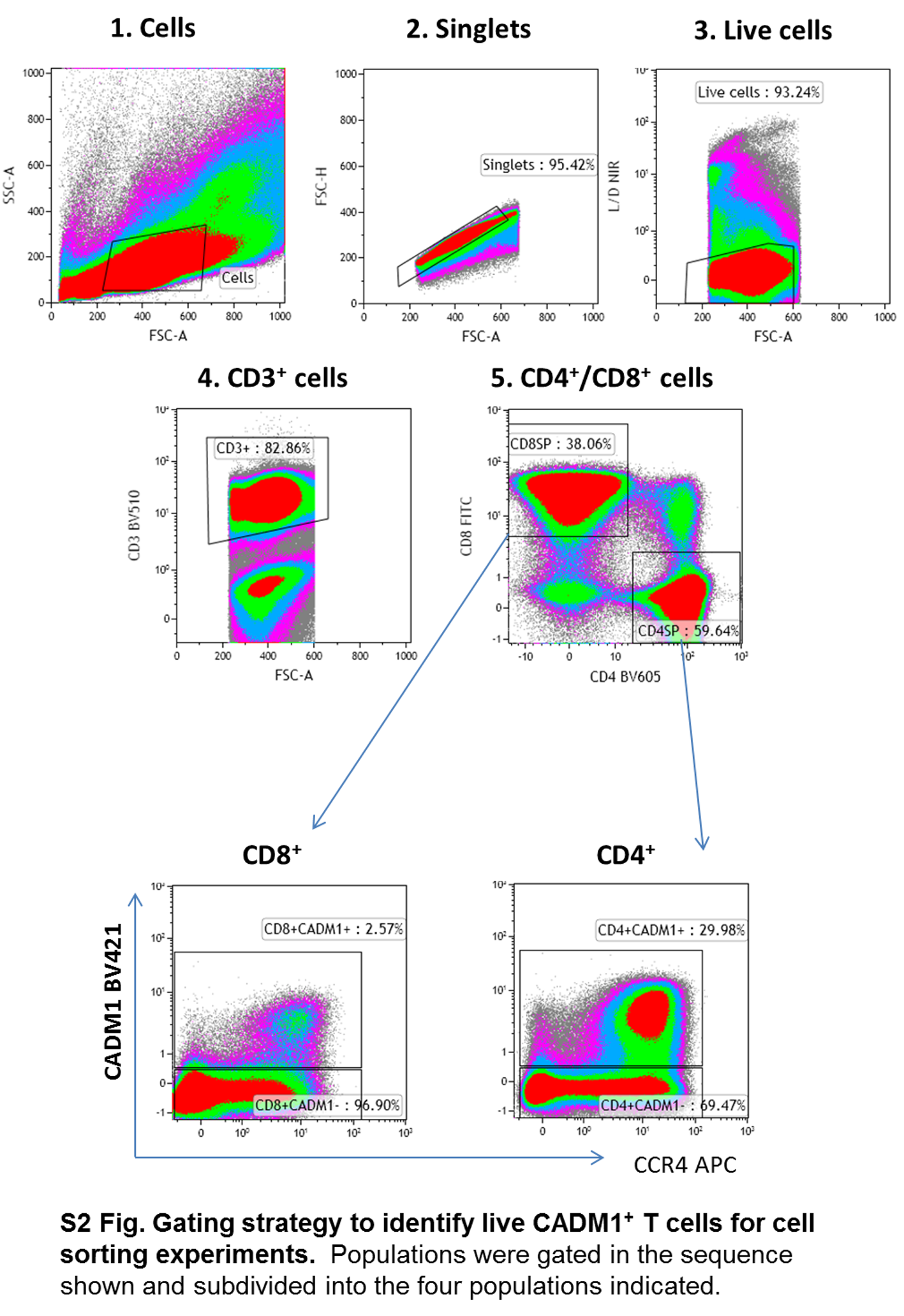

Supplement: S2 Fig — (TIF) [file ppat.1005560.s003.tif]

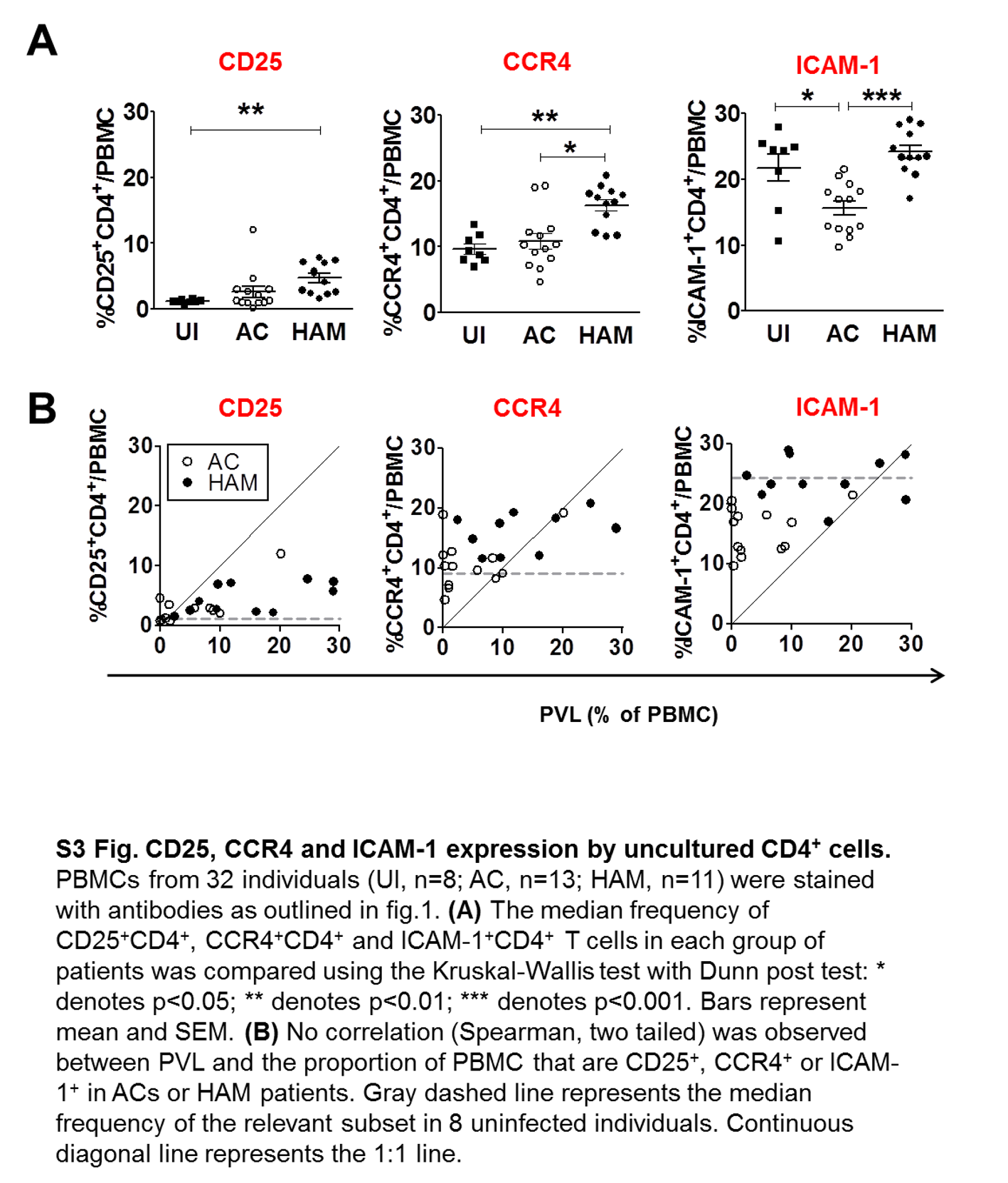

Supplement: S3 Fig — (A) The median frequency of cells in each group of patients. (B) PVL versus frequency of cells in each subset. (TIF) [file ppat.1005560.s004.tif]

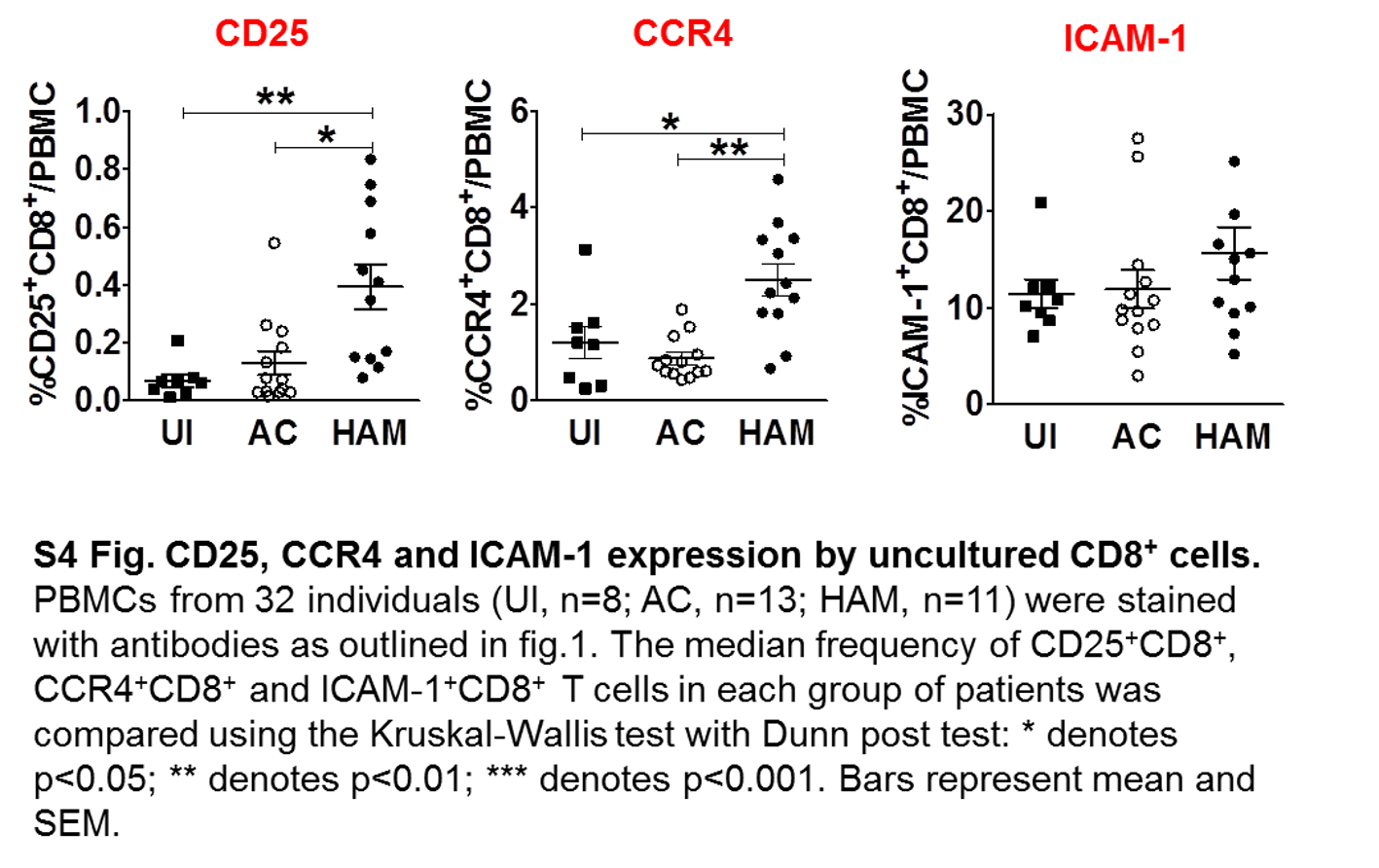

Supplement: S4 Fig — (TIF) [file ppat.1005560.s005.tif]

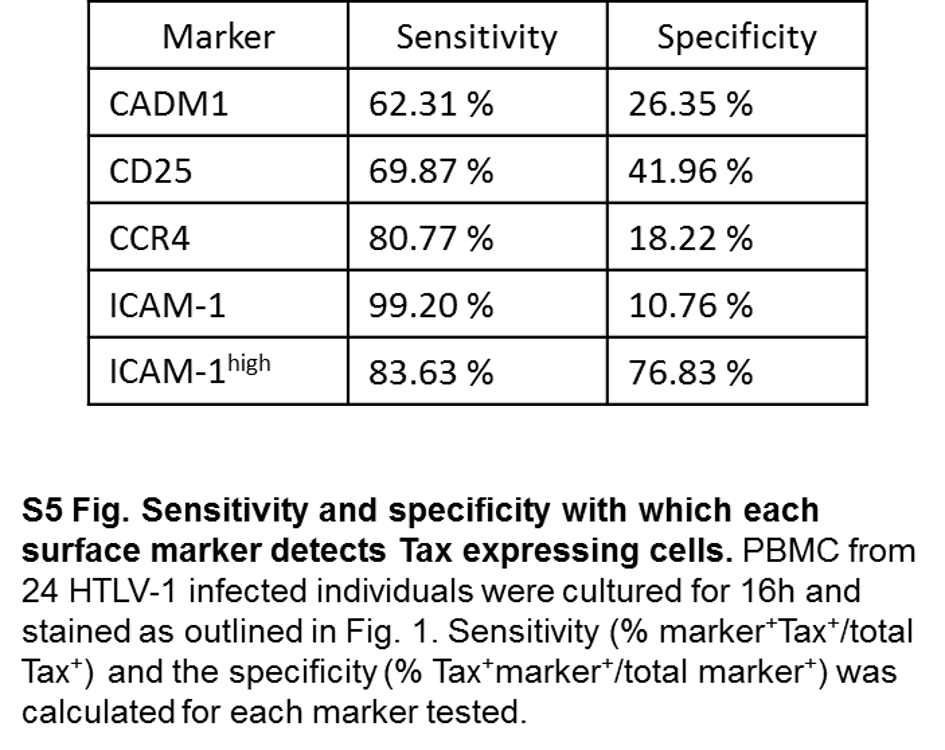

Supplement: S5 Fig — (TIF) [file ppat.1005560.s006.tif]

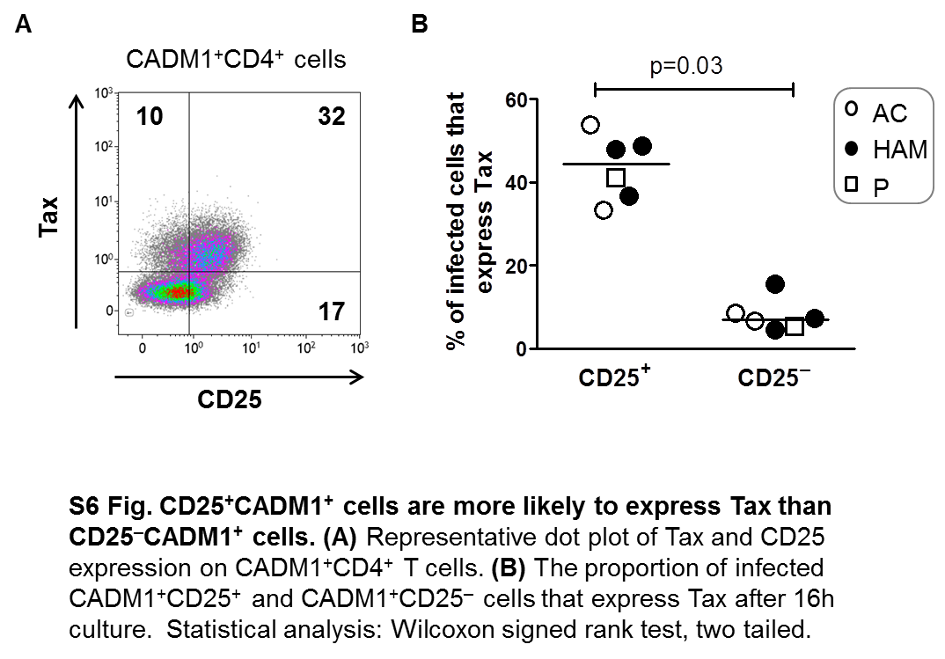

Supplement: S6 Fig — (A) Representative dot plot. (B) The proportion of CADM1+CD25+ and CADM1+CD25– cells that express Tax after 16h culture. (TIF) [file ppat.1005560.s007.tif]

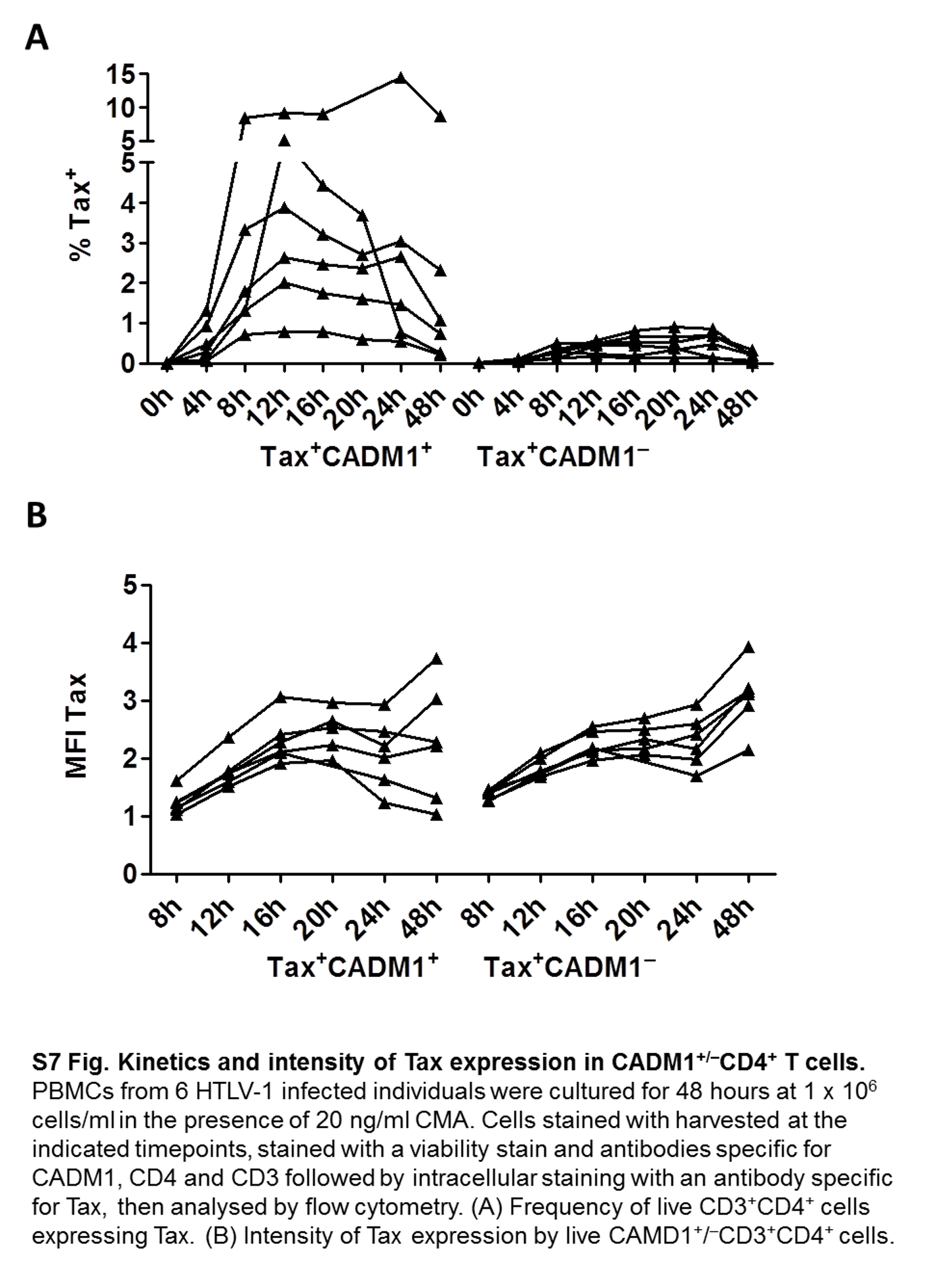

Supplement: S7 Fig — (A) Frequency of live CD3+CD4+ cells expressing Tax. (B) Intensity of Tax expression by live CAMD1+/–CD3+CD4+ cells (TIF) [file ppat.1005560.s008.tif]

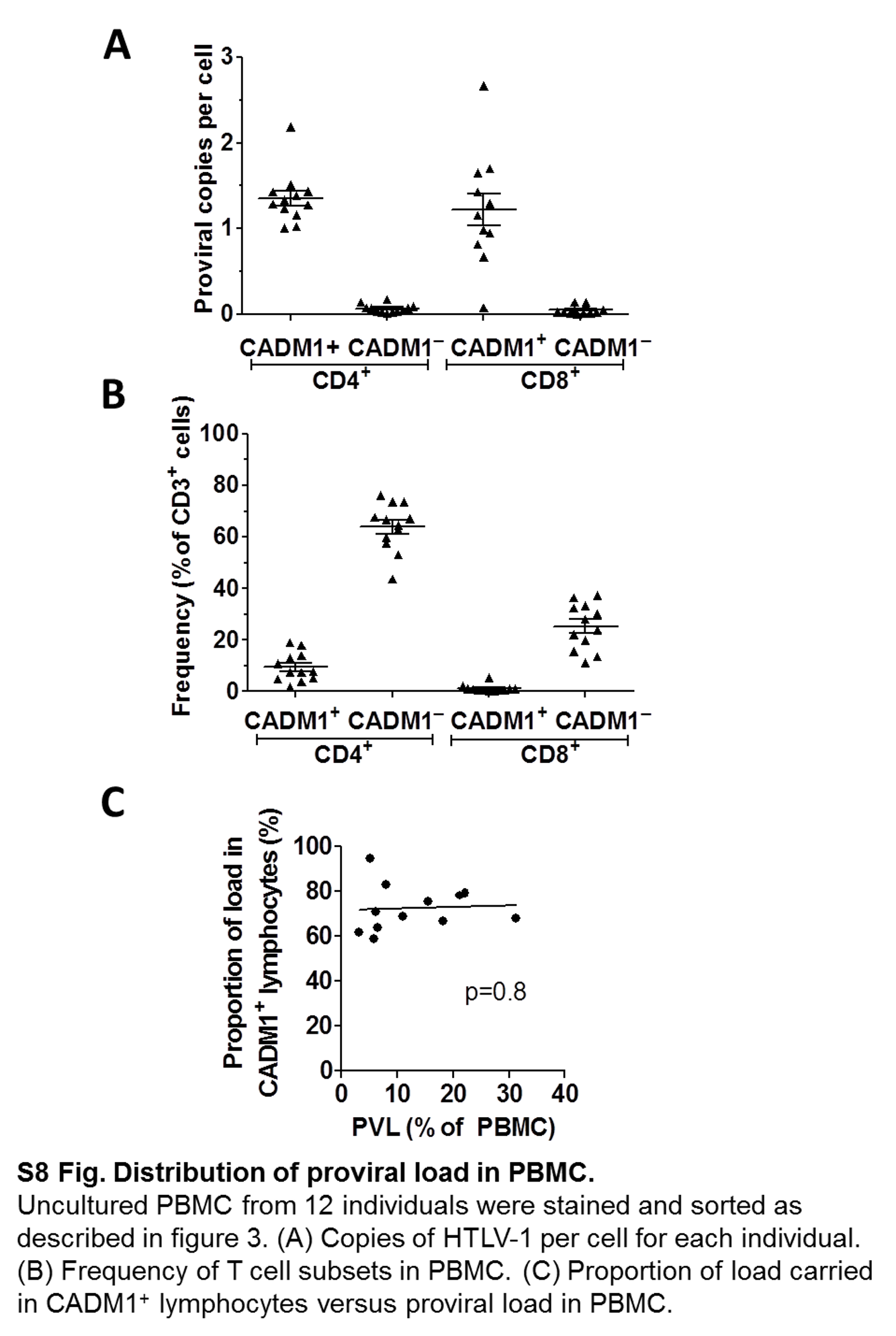

Supplement: S8 Fig — (A) Copies of HTLV-1 per cell for each individual. (B) Frequency of T cell subsets in PBMC. (C) Proportion of load carried in CADM1+ lymphocytes versus proviral load in PBMC. (TIF) [file ppat.1005560.s009.tif]

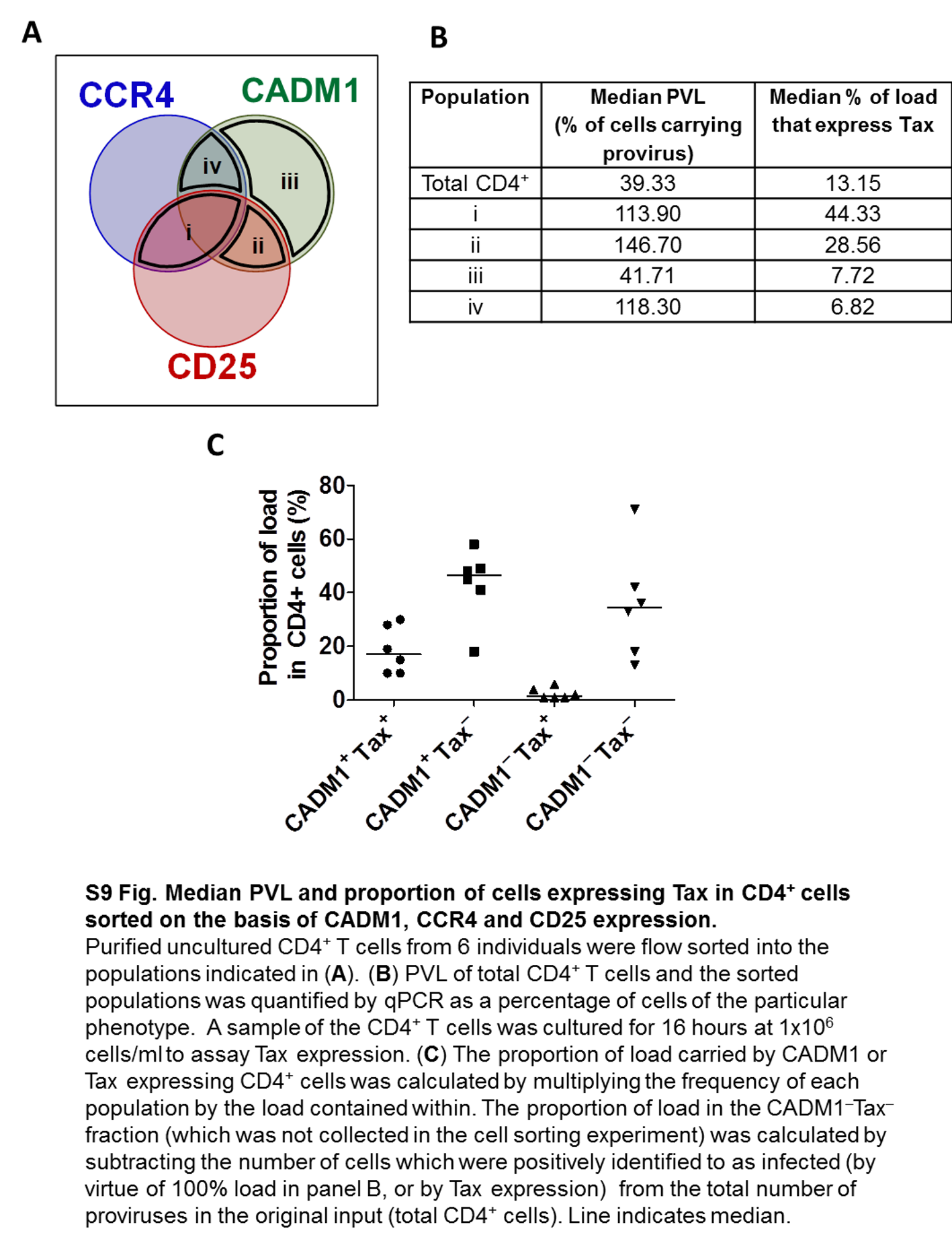

Supplement: S9 Fig — (A) Sorting strategy. (B) PVL of total CD4+ T cells and sorted populations. (C) The proportion of load carried by CADM1 or Tax expressing CD4+ cells. (TIF) [file ppat.1005560.s010.tif]
